# A twin study of body mass index and dental caries in childhood

**DOI:** 10.1038/s41598-020-57435-7

**Published:** 2020-01-17

**Authors:** M. J. Silva, N. M. Kilpatrick, J. M. Craig, D. J. Manton, P. Leong, H. Ho, R. Saffery, D. P. Burgner, K. J. Scurrah

**Affiliations:** 1 Facial Sciences, Murdoch Children’s Research Institute, Parkville, Australia; 2 Department of Paediatrics, University of Melbourne, Melbourne, Australia; 3 Inflammatory Origins, Murdoch Children’s Research Institute, Parkville, Australia; 4 Melbourne Dental School, University of Melbourne, Melbourne, Australia; 5 Centre for Molecular and Medical Research, School of Medicine, Faculty of Health, Deakin University, Victoria, Australia; 6 Molecular Epidemiology, Murdoch Children’s Research Institute, Parkville, Australia; 7 Centrum voor Tandheelkunde en Mondzorgkunde, Universitair Medisch Centrum Groningen, Rijksuniversiteit Groningen, Groningen, The Netherlands; 8 Epigenetics, Murdoch Children’s Research Institute, Parkville, Australia; 9 Department of Paediatrics, Monash University, Melbourne, Australia; 10 Infectious Diseases, Royal Children’s Hospital, Melbourne, Australia; 11 School of Population and Global Health, University of Melbourne, Melbourne, Australia

**Keywords:** Dental caries, Paediatric research

## Abstract

Sub-optimal nutrition and dental caries are both common with significant short and long-term implications for child health and development. We applied twin statistical methods to explore the relationship between body mass index (BMI) and dental caries. We measured BMI at 18 months and six years of age and cumulative dental caries experience at six years in 344 twin children. Dental caries in primary teeth was categorised into ‘any’ or ‘advanced’ and BMI was analysed as both a continuous and categorical variable. Statistical analyses included multiple logistic regression using generalized estimating equations and within/between-pair analyses. There was no association between BMI and ‘any’ dental caries experience at either time-point, neither overall nor in within/between pair analyses. However, ‘advanced’ dental caries at six years was associated with a within-pair difference in BMI of −0.55 kg/m^2^ (95% CI −1.00, −0.11, p = 0.015). A within-pair increase of 1 kg/m^2^ in BMI was associated with a lower within-pair risk of advanced dental caries (OR 0.68, 95% CI 0.52, 0.90, p = 0.007). These findings reveal a possible causal relationship between lower BMI and dental caries. As dental outcomes were only measured at one time point, the direction of this potentially causal relationship is unclear.

## Introduction

Poor nutrition and dental caries are both common with significant short and long-term implications for child health and development^1,2^. Understanding how growth and dental caries, a common childhood condition are linked could inform broader risk factor-based preventive strategies, and also facilitate cross-disciplinary and collaborative approaches between public health, dental and medical specialists involved in the care of children^3^. Despite a number of systematic reviews, the association between BMI and dental caries remains unclear^4–7^. The relationship may be bidirectional, as both altered BMI and dental caries might be perceived be an exposure or an outcome. In addition to common risk factors (such as dietary sugar intake)^3^, there are also several plausible (albeit speculative) biological causal pathways between BMI and dental caries. Additionally, the relationship may be non-linear and depend on the study setting and socioeconomic profile of participants, with higher levels of dental caries reported in both underweight children in low to middle income countries (LMICs) and in obese children in high-income countries^4,6^.

Observational studies of twins can provide a valuable tool to explore complex relationships such as that between BMI and dental caries, because they remove much of the bias resulting from confounding^8^. As twins share genetic variation (100% for MZ twins and 50% for DZ twins) and many (but not all) environmental factors, such confounding variables can be controlled for, even if they are not directly measured. Therefore, demonstrating robust differences in outcomes between exposed and non-exposed twins may suggest consistency with causal relationships^9^. Within/between-pair analyses can be undertaken to separate the effect of shared (between-pair) and non-shared (within-pair) measured influences^10^.

The aim of this study is to use twin cohort data to evaluate the associations, including potential causal relationships, between BMI and dental caries. We tested three hypotheses: (1) BMI at 18 months is a predictor of dental caries at six years; (2) BMI at six years is a predictor of dental caries at six years and (3) dental caries at six years is a predictor of BMI at six years. Hypotheses 2 and 3 reflect the potential bi-directional association between cross-sectional observations at six years.

## Results

**Figure 1:**
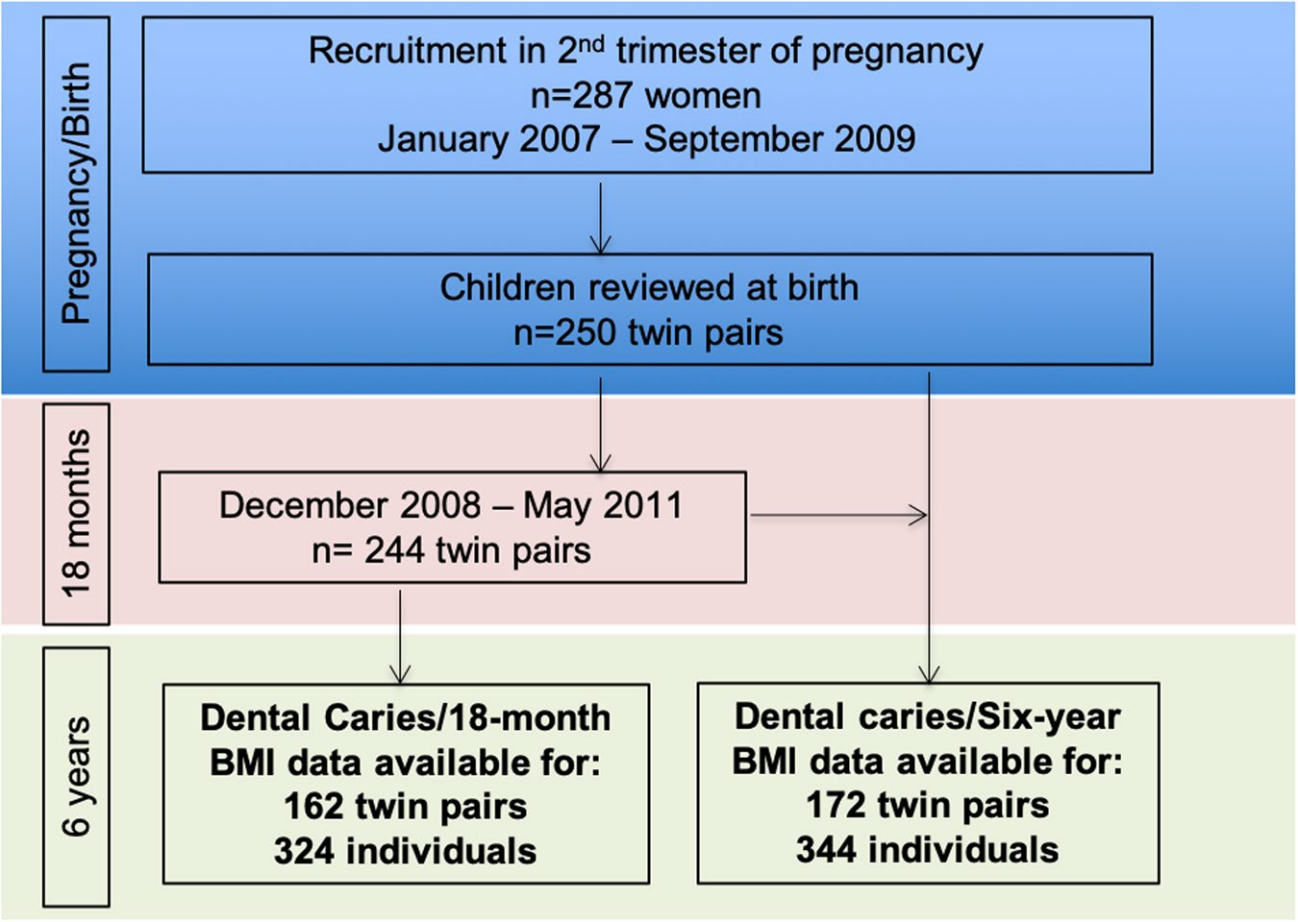
Cohort recruitment and retention.

A total of 344 children (from 101 DZ and 71 MZ pairs; 69% of the original birth cohort) had dental examinations and anthropometry measured at the six-year assessment and 324 children had complete data from the six-year and 18-month assessments (Fig. 1). The mean age of the children at the six-year assessment was 6.7 years (range 6.0–9.0, SD 0.64) and 186 (54.1%) were female (Table 1). The mean SEIFA score for the cohort was 1018.2 (SD 55.0), indicating the sample had higher SES than the general Australian population.

**Table 1:** Demographic details, dental caries, BMI and potential confounders in participants of six-year assessment.

Variable	Dental Study Mean [SD]; or N (%) n = 344
Monozygotic twin	142 (41.3)
Female	150 (45.9)
Age	6.7 [0.6]
**Sugar intake**	
Low	83 (24·8)
Medium	214 (64.1)
High	38 (11·1)
**Brushing frequency**	
2 times-a-day	204 (61.8)
Once-a-day	106 (31·8)
Once every 2–4 days	21 (6.4)
SEIFA at six years	1018.2 [55.0]
Any dental caries	111 (32.3)
Advanced dental caries	83 (24.1)
BMI (kg/m^2^) at 18-month assessment	16.6 [1.3]
**BMI at six-year assessment**	
BMI (kg/m^2^)	15.8 [1.8]
Underweight	36 (10.5)
Healthy BMI	256 (74.4)
Overweight	40 (11.6)
Obese	12 (3.5)

A total of 111 (32.3%) children had ‘any caries’ and a total of 83 (24.1%) children had ‘advanced caries’. The mean BMI at the 18-month assessment was 16.6 kg/m^2^ (1.3) and −0.30 (SD 1.1) standardised. The mean BMI at the six-year assessment was 15.8 kg/m^2^ (SD 1.8) and 0.10 (SD 1.1) standardised, with 38 children (11.0%) underweight and 52 children (15.1%) overweight or obese.

### Hypothesis 1: BMI at 18 months as exposure and dental caries at six years as outcome

There were no associations between BMI at 18 months and ‘any’ or ‘advanced’ dental caries at six years (Table 2). Within/between pair analysis also did not identify an association between BMI at 18 months and ‘any’ or ‘advanced’ caries at six years.

**Table 2:** The association between BMI at the 18-month assessment and ‘any’ and ‘advanced’ dental caries.

Factor	Any caries				Advanced caries			
	Unadjusted OR (95% CI)	Unadjusted P-value	Adjusted OR (95% CI)	Adjusted P-value	Unadjusted OR (95% CI)	Unadjusted P-value	Adjusted OR (95% CI)	Adjusted P-value
	(n = 324)	(n = 324)	(n = 306)	(n = 306)	(n = 324)	(n = 324)	(n = 306)	(n = 306)
BMI (kg/m^2^)	1.05 (0.86, 1.26)	0.62	1.02 (0.82, 1.26)	0.88	1.06 (0.88, 1.28)	0.53	1.04 (0.84, 1.28)	0.75
**Within/Between-pair analysis**								
Within-Pair	1.09 (0.77, 1.54)	0.62	1.02 (0.72, 1.45)	0.92	0.88 (0.63, 1.21)	0.42	0.82 (0.58, 1.16)	0.27
Between-Pair	1.02 (0.81, 1.29)	0.85	1.00 (0.79, 1.26)	0.99	1.19 (0.92, 1.53)	0.18	1.14 (0.88, 1.47)	0.33

^*^Within pair analyses adjusted for sex and diet, overall analysis adjusted for sex, age, community water fluoridation, sugar consumption, tooth brushing and SEIFA, **The outcomes when using BMI standardised for age and sex were similar to those when raw BMI scores were used.

### Hypothesis 2: BMI at six years as exposure and dental caries at six years as outcome

Overall, there was no evidence of an association between the exposure BMI at six years (either as a continuous or categorical variable) and ‘any’ dental caries (Table 3). Adjusting for potential confounders including age, sex, sugar consumption, tooth-brushing frequency, SEIFA at six years of age, and community water fluoridation did not alter the results. Within/between pair analysis did not identify an association between BMI at six-years (exposure) and ‘any caries’ at six-years (outcome), adjusted OR 0.91 (95% CI 0.72, 1.17, p = 0.47) for a within pair difference of 1 kg/m^2^ and 1.13 (95% CI 0.94, 1.36, p = 0.19) for a between-pair difference of 1 kg/m^2^ (Table 3).

**Table 3:** BMI at six-year assessment (kg/m^2^) as predictor and dental caries at six-year assessment as outcome.

Factor	Any caries				Advanced caries			
	Unadjusted OR (95% CI)	Unadjusted P-value	Adjusted OR (95% CI)^*^	Adjusted P-value^*^	Unadjusted OR (95% CI)	Unadjusted P-value	Adjusted OR (95% CI)^*^	Adjusted P-value^*^
	(n = 344)	(n = 344)	(n = 325)	(n = 325)	(n = 344)	(n = 344)	(n = 325)	(n = 325)
BMI (kg/m^2^)	1.09 (0.94, 1.26)	0.27	1.04 (0.90, 1.21)	0.58	1.04 (0.89, 1.23)	0.62	1.03 (0.88, 1.20)	0.74
**BMI categories**								
Normal BMI	1	Ref	1	Ref	1	Ref	1	Ref
Underweight	1.15 (0.56, 2.37)	0.70	1.10 (0.49, 2.44)	0.82	0.93(0.42, 2.05)	0.86	0.86 (0.35, 2.12)	0.74
Overweight or obese	1.16 (0.57, 2.38)	0.68	0.99 (0.45, 2.18)	0.99	1.11 (0.50, 2.47)	0.80	1.05 (0.46, 2.41)	0.91
**Within/Between-Pair analysis**								
Within-pair	0.96 (0.75, 1.22)	0.73	0.91 (0.72, 1.17)	0.47	0.72 (0.55, 0.94)	0.02	0.68 (0.52, 0.90)	0.01
Between-pair	1.15 (0.96, 1.39)	0.13	1.13 (0.94, 1.36)	0.19	1.21 (0.99, 1.47)	0.06	1.20 (0.99, 1.46)	0.06

^*^Within pair analyses adjusted for sex and sugar consumption, overall analysis adjusted for sex, age, community water fluoridation, sugar consumption, tooth brushing and SEIFA, **The outcomes when using BMI standardised for age and sex were similar to those when raw BMI scores were used.

Overall, there was no evidence of associations between the exposure BMI at six years (either as a continuous or categorical variable) and ‘advanced’ dental caries, including after adjustment for potential confounders (Table 3). However, there was strong evidence of an association between ‘advanced’ caries and within-pair BMI difference (per kg/m^2^) (adjusted OR 0.68, 95% 0.52, 0.90, p = 0.01) (Table 3). The lower risk of ‘advanced caries’ associated with higher BMI (per kg/m^2^) within twin pairs did not differ by zygosity (p = 0.27), OR 0.46 (95% CI 0.21, 1.02, p = 0.06) in MZ twin pairs; OR 0.74 (95% CI 0.56, 0.98, p = 0.04) in DZ twin pairs.

### Hypothesis 3: Dental caries at six years as exposure and BMI at six years as outcome

Overall, there was no evidence of an association between ‘any’ dental caries (exposure) and the outcome, BMI at six years (either as a continuous or categorical variable), including after adjusting for confounders (Table 4). Within/between-pair analysis also failed to find an association, with a within-pair difference of −0.16 kg/m^2^, 95% CI −0.61, 0.28, p = 0.47 and a between-pair difference of 0.41 (95% CI −0.23, 1.07, p = 0.22) (Table 4).

**Table 4:** Dental caries as predictor and BMI (kg/m^2^) at six-year assessment as outcome *Within/Between pair analyses adjusted for sex and sugar consumption, overall analysis adjusted for sex, age, community water fluoridation, sugar consumption, tooth brushing and SEIFA.

	Unadjusted BMI kg/m^2^ (95% CI)	Unadjusted P-value	Adjusted BMI kg/m^2^ (95% CI)*	Adjusted P-value
‘Any’ caries	0.12 (−0.24, 0.49)	0.51	−0.03 (−0.39, 0.33)	0.86
Within-pair	−0.07 (−0.51, 0.36)	0.74	−0.16 (−0.61, 0,28)	0.47
Between-pair	0.51 (−0.15, 1.16)	0.13	0.41 (−0.23, 1.07)	0.22
‘Advanced’ caries	−0.14 (−0.51, 0.24)	0.47	−0.28 (−0.63, 0.77)	0.12
Within-pair	−0.50 (−0.93, −0.07)	0.02	−0.55 (−1.00, −0.11)	0.02
Between-pair	0.70 (−0.05, 1.46)	0.07	0.61 (−0.12, 1.33)	0.10

Overall, there was also no evidence of associations between ‘advanced’ dental caries (exposure) and outcome, BMI, at six years (as a continuous variable), including after adjustment for confounders (Table 4). However, within pairs, the twin with ‘advanced caries’ had a lower BMI at six years- of- age of −0.55 kg/m^2^ (95% CI −1.00, −0.11, p = 0.02, Table 4) after adjusting for non-shared factors, sugar consumption and sex when compared with the twin with minimal or no caries. There were small but non-significant differences between MZ and DZ twin pairs, at −0.44 kg/m^2^ (95% CI −1.01, −0.13, p = 0.13) and −0.63 kg/m^2^ (95% CI −1.26, 0.01, p = 0.05) respectively.

## Discussion

Our longitudinal twin study of Australian children found no overall association between BMI (at 18 months and six years of age) and ‘any’ or ‘advanced’ dental caries at six years of age, after adjusting for known confounders. However, in within/between-pair analyses that adjust more broadly for potential known and unknown confounders, lower BMI was associated with advanced caries, consistent with a causal relationship.

Unravelling the relationship between BMI and dental caries is challenging. Four systematic reviews have yielded conflicting results, with three reporting no overall association and one finding higher rates of dental caries in obese children^4–7^. Although differences in study methodology may contribute to inconsistent findings, the study setting and age of participants may also influence the results. In one of these reviews, the authors suggest that different factors are likely to influence the BMI and dental caries relationship in different socio-economic strata^6^. For example in LMICs it is possible that the degree of underweight and malnutrition is sufficient to impact on the development of teeth resulting in poorly formed or hypomineralised dental enamel which is a recognised caries risk factor^11^. Such severe nutritional deficiencies are unlikely in paediatric populations in high-income countries. Alternatively, in high-income countries with high rates of obesity, excess dietary sugar intake is likely to confound any potential causal relationship between BMI and caries. The present study sample was relatively affluent from a high-income country. Our finding of an association between low BMI and dental caries has been observed in both high E.g. Sweden and Kuwait and LMICs such as Bangladesh and Thailand^12–15^.

As the direction of the relationship between BMI and dental caries is unclear, it is pertinent to consider the underlying biological mechanisms that might explain how ‘advanced’ dental caries may lead to lower BMI as well as the alternative, albeit less plausible hypothesis of how low BMI may lead to high levels of dental caries. Compromised ability to chew or bite foods may explain how ‘advanced’ (but not ‘any’) dental caries contribute to a lower BMI as teeth with larger “advanced” lesions may have a higher impact than initial lesions. Difficulty eating is commonly reported in children with dental caries and worsens as the severity of dental caries increases^16,17^. This hypothesis is further supported by the catch-up growth and weight gain reported in underweight children after treatment of teeth with carious lesions, suggesting that dental caries can impede growth and development in children^18^. Although more speculative, another potential mechanism may be related to underlying biological processes. For example, chronic inflammation secondary to infection caused by dental caries has been linked with anaemia due to cytokine mediated inhibition of erthyropoieses^19^. The association with advanced, but not any caries, suggests a threshold level of dental caries that impacts on BMI, with early, superficial carious lesions having little effect on quality and quantity of nutritional intake.

Although arguably less plausible, diet may also mediate the relationship between low BMI and increased dental caries risk. It is possible that sugary foods and drinks can be used to promote weight gain in thin, underweight children, although dietary sugar consumption was included in the statistical models and this is likely to only apply to a small group of children^20^. Alternatively, underweight children may have nutritional deficiencies that predispose them to developmental dental defects (such as poorly mineralised enamel) which in turn increase dental caries risk^4^. However, as tooth formation occurs very early in life, and in the case of the primary dentition antenatally, for this mechanism to impact, these children would have to be underweight (and nutritionally deficient) in early infancy or even in utero. However, our study failed to find any such association between BMI at 18-months and dental caries at six years, suggesting this is less likely.

As the direction and mechanism explaining the within-pair links between low BMI and dental caries remains unclear, it is possible that there may be several different, and perhaps even contradictory pathways. In addition to the factors discussed above, as the association was evident within pairs, the underlying mechanism(s) could also be due to additional unknown/unmeasured non-shared factors that have not been fully accounted for in this study. Therefore, further longitudinal twin studies, exploring non-shared factors including biological pathways such as inflammation and social factors like temperament between twins may provide valuable insight into the links between BMI and dental caries.

Although within-pair differences were noted between BMI and advanced dental caries, there was no association in the overall multiple regression model, after adjusting for known confounders. However, multiple regression is not as powerful as within-pair analyses in adjusting for confounders as it only accounts for known and measured covariates. Twin studies are particularly advantageous in exploring complex associations and causal relationships because they can adjust for both known and unknown, measured and unmeasured potential shared confounders^9^. The nature and contribution of these factors may well be different in different settings and may explain why both high and low BMI have been associated with dental caries in other studies. Clinicians involved in the care of young children should be cautious about presuming that a child with low BMI is at heightened caries risk or *vice versa*, because of the likely contribution of numerous environmental factors at the population, family and individual level.

Nevertheless, the within-pair association between BMI and dental caries identified in our study highlights the importance of considering oral health as part of (and not separate from) general health^21^. Although the direction and mechanism of the association remains unclear, both adverse BMI and dental health are likely to have many shared pathways^22^. Integrating assessment of oral health into longitudinal cohort studies, including ante-natal exposure data, from as early as one year-of-age, is essential to further understand how oral and systemic health interact across the life course. Additionally, the possible links between advanced dental caries and growth has important implications for global health, particularly as wasting (low weight for-height) continues to affect 52 million children worldwide^23^. Prevention of dental caries will benefit from broader, common risk factor approaches that target children early in life to ensure optimal health in early childhood for long-term health and well-being^3^.

The interpretation of BMI in young children is complicated by normal, developmental physiological changes that occur with growth^24^. Using standardised measures, as in our study, overcomes some of these issues. Additionally, BMI does not directly measure adiposity. However, it is an easily measured and calculated metric that is closely correlated with more granular adiposity measures (that come at a cost in terms of time, economic and participant burden), such as dual energy X-ray absorptiometry, hydrostatic weighing, air-displacement plethysmography, isotope dilution, bioelectrical impedance analysis and skin-fold thickness measurements^25^.

The study addresses many limitations of previous studies, with robust measurement of dental caries using ICDAS criteria to record early carious lesions as well as advanced disease, and measurement of BMI at multiple time points. In addition, this is the first study to apply twin analyses to explore the relationship between dental caries and BMI and highlights the contribution of twin studies in understanding multifactorial conditions, with potential generalisability to the broader population. However, we acknowledge some limitations. Although there was high participant retention, the sample size limited the power and precision of some estimates. It is important to note that there was limited statistical power to evaluate an association with a categorical classification of abnormal BMI. Further, the lack of longitudinal dental data, and in particular the lack of caries data at 18 months of age, limits our ability to establish the direction of the potential causal relationship. More sensitive measures of adiposity, taken at more regular intervals would provide further insight into how variations in growth and adiposity are related to dental caries, and may also inform causal inferences. Additionally, dietary sugar intake is an important confounder that is difficult to measure particularly when, as in this study, it is based upon parental report only. Finally, only a single, area-level indicator of SES was used, and may not reflect individual or household SES, which may be more important in the context of oral health.

## Conclusion

Within and between twin analyses adjusted for known and unknown confounders found that within pairs advanced dental caries was associated with lower BMI at six years. Twin studies and analyses provide unique insight into the determinants of caries risk and further prospective longitudinal cohort studies, from both developing and developed countries, aimed at understanding the direction of any causal relationships and the underlying mechanisms and influences in both singletons and twins are warranted.

## Methods

This cross-sectional study is part of The Peri/postnatal Epigenetic Twins Study (PETS), a longitudinal birth cohort of 250 mothers and their twin children, established in 2007^26^. Initial recruitment was in early-mid pregnancy. Data regarding the health and lifestyle of the mothers were collected through pregnancy. To date, the children have participated in three reviews; at birth, 18 months and six years of age.

The current study draws on BMI data collected at 18 months and six years and dental data collected at six years. Participant weight was recorded in kilograms with a digital weight scale, in light clothing. Participant standing height was recorded in centimetres using a stadiometer with a fixed vertical backboard and an adjustable headpiece. Dental caries was recorded using the International Caries Detection and Assessment System (ICDAS), which quantifies carious lesions from early to large cavitated lesions with significant destruction of tooth structure^27^. Details regarding dental examination kappa scores and examination procedures have been reported elsewhere^28^. Weight and height at 18-months were measured at the research institute or children’s homes. Dental examinations and height and weight measurement at six-years were performed at the research facility. A minority of assessments, for participants unable to attend the research facility, were conducted at home by the same research team using the same protocols.

Zygosity was determined for all same-sex twin pairs by chorionicity and genetic analysis of a 12-marker microsatellite test using DNA from cord and/or buccal samples^29^. Children’s sugar consumption was classified into low, medium or high by the reported weekly frequency and number of sweets, including chocolate, lollies, sweet biscuits, soft drink, ice cream and sweet spreads^28^. The socioeconomic status (SES) of participants at birth was obtained by linking maternal postcodes in early pregnancy to the Index of Relative Socio-Economic Disadvantage, one of the Socio-economic indexes for areas (SEIFA) developed by the Australian Bureau of Statistics based on 2006 census data^30^. The SEIFA index is standardised to a mean of 1000 and a standard deviation (SD) of 100. Increasing SEIFA score indicates lower socio-economic disadvantage^31^. Access to fluoridated town water was determined from the Victorian Department of Health and Human Services website by using participants’ current postcode at age six years^32^.

Ethics approval was granted by the Royal Children’s Hospital (Melbourne, Australia) Human Research Ethics Committee (33174A) and informed consent was obtained from a parent/guardian. Research, including clinical dental examinations adhered to all relevant guidelines and regulations.

### Data analysis

The study data were collected and managed using REDCap electronic data capture tools^33^ and analysed using STATA 15 (StataCorp. 2017. *Stata Statistical Software: Release 15*. TX, USA). Two binary variables, the presence or absence of ‘any caries’ (including all carious lesions with ICDAS caries codes 1 and above. and/or past treatment) and the presence or absence of ‘advanced caries’, (established carious lesions with ICDAS codes 4 to 6 and/or past treatment) were derived from the ICDAS index as reported previously^28^. The status of the pulp was not assessed.

BMI was calculated using standing height and weight and standardised for age and sex using the “ZANTHRO” STATA package with the UK growth charts as ref. ^34^. Categorical variables for underweight, overweight and obese were calculated using the “zbmicat” STATA function, which uses cut-offs based on age, sex, and BMI^35^. These correspond to equivalent adult BMI cut-off points endorsed by the World Health Organization of BMI < 25 kg/m^2^ for normal weight, BMI 25–29.99 kg/m^2^ for overweight, and BMI ≥ 30 kg/m^2^ for obesity.

To assess association between BMI and dental caries, logistic and linear regression models were fitted, using a generalized estimating equations (GEE) approach to adjust for twin correlation. To explore potential causal relationships, we fitted within- and between-pair models using GEE for each of the three hypotheses^36^. The results of the GEE analysis were checked using post-estimation diagnostics. Both raw and standardised BMI were used in these analyses.
